# Case Study of a Parotid Gland Adenocarcinoma Dedifferentiated from Epithelial-Myoepithelial Carcinoma

**DOI:** 10.1155/2014/629054

**Published:** 2014-09-09

**Authors:** Salih Aydın, Umit Taskin, Kadir Ozdamar, Kadir Yücebas, Mehmet Sar, Mehmet Faruk Oktay, Ümit Seza Tetikkurt

**Affiliations:** ^1^Department of Otolaryngology, Bağcılar Education and Research Hospital, Istanbul, Turkey; ^2^Department of Pathology, Bağcılar Education and Research Hospital, Istanbul, Turkey

## Abstract

Dedifferentiation is defined as high-grade malignant tumor development out of a low-grade malignant tumor. We present an adenocarcinoma tumor of the parotid gland that was dedifferentiated from a low-grade epithelial-myoepithelial carcinoma and was followed up for 3 years. Our patient, a 46-year-old female, presented with a left parotid mass of 20-year duration. Histopathologic results showed that there was only one area of typical epithelial-myoepithelial carcinoma, with foci of poorly differentiated adenocarcinoma (not otherwise specified; NOS) and clear cytoplasm in the parotid gland. Immunohistochemical staining results showed SMA (+), P63 (+), CK8 (+), and S100 (+) on epithelial cells. A review of the literature revealed 22 previously reported cases of dedifferentiated epithelial-myoepithelial carcinoma. In these cases, the malignant tumors that dedifferentiated from epithelial-myoepithelial carcinoma were adenoid cystic carcinoma, actinic cell carcinoma, polymorphous low-grade adenocarcinoma, mucoepidermoid carcinoma, and intraductal carcinoma. In our case, the malignant tumor that dedifferentiated from the epithelial-myoepithelial carcinoma was a poorly differentiated adenocarcinoma. Histopathological results showed that metastases were not seen in the neck-dissection material. As a result, our case will make a contribution to the literature in terms of prognosis, because there are very few reported cases of dedifferentiated adenocarcinoma development arising from epithelial-myoepithelial carcinoma.

## 1. Introduction

Epithelial-myoepithelial carcinomas account for less than 1% of all salivary gland malignancies [1]. Epithelial-myoepithelial carcinomas are classically low-grade malignancies. As per the literature, they involve the parotid gland most frequently and the minor salivary glands rarely [1].

High-grade malignant tumor foci may be differentiated from low-grade malignancies in the salivary glands. The main reason for this differentiation is not known [2, 3]. There is no histopathologic evidence of this differentiation. This process is known as “high-grade transformation” and “hybrid tumor” in the literature, but “dedifferentiation” is the most current definition in general use [2–4]. In these tumors, the clinical presence of neck metastasis, distant metastasis, and local recurrence may be considered an indicator of poor prognosis [3]. On the other hand, the prognosis of dedifferentiated tumors is not accurately known because of limited cases in the literature. We have presented a case of dedifferentiated carcinoma of the parotid gland that was followed up for 3 years.

## 2. Case Presentation

**Figure 1:**
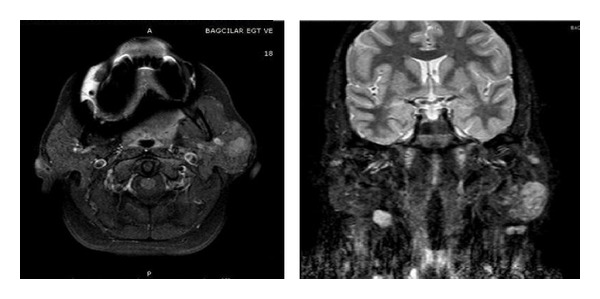
MRI demonstrates solid mass lesion in left parotid gland. Fat-suppressed T2-weighted images show mild hyperintensity, T1-weighted images show hypointensity, and postcontrast images show low contrast enhancement.

**Figure 2:**
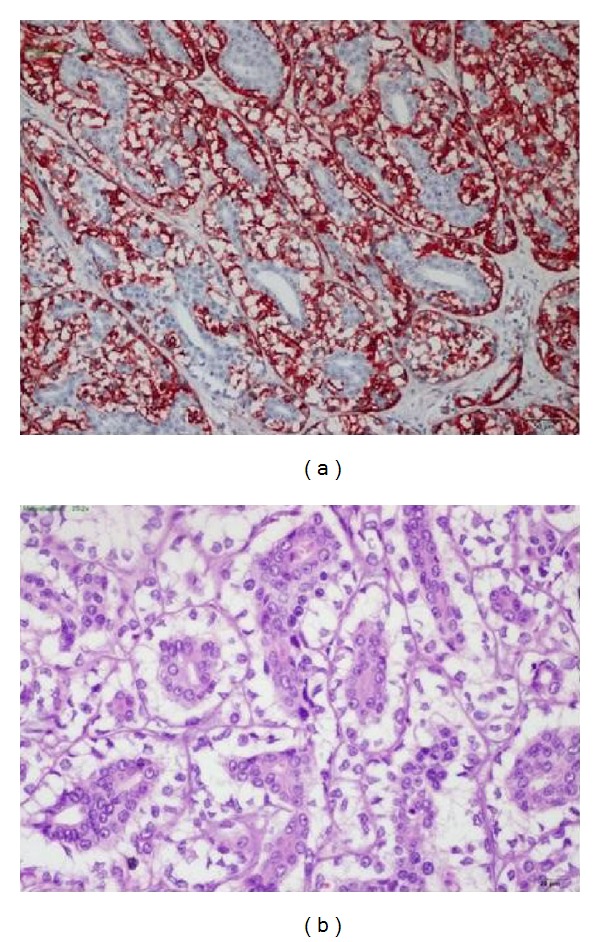
Myoepithelial cells with smooth muscle actin (SMA) positivity on immunohistochemistry ×200 (a). There are bilayered tumor cells in the basal layer, the outer myoepithelial cells have a clear appearance, and the inner epithelial cells have lumen formation with hematoxylin and eosin ×200 (b).

**Figure 3:**
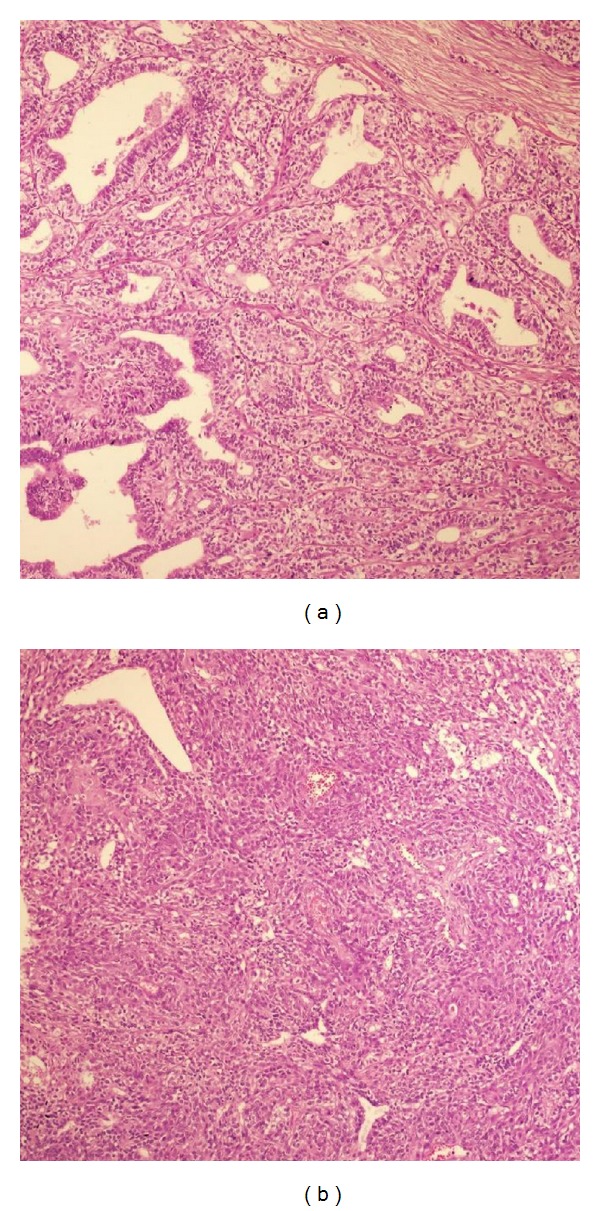
Epithelial-myoepithelial carcinoma and adenocarcinoma (NOS) in the same aspect, hematoxylin and eosin ×100 (a). Adenocarcinoma appearance which had solid structure and rare glandular structures together, hematoxylin and eosin ×100 (b).

Our patient is a 46-year-old female who presented with a left parotid mass of 20-year duration that was increasing in size over a 4-year period. Upon examination, there was a 4 × 5 cm firm, painless, mobile mass, and facial nerve function was intact. A 3.5 cm solid lobulated mass lesion with a soft edge property in the left parotid gland was identified on MRI (fat-suppressed T2-weighted images showed mild hyperintensity, T1-weighted images showed hypointensity, and postcontrast images showed low contrast enhancement) (Figure 1). Fine needle aspiration biopsy revealed benign mixed tumor. Due to these findings, a left superficial parotidectomy was performed. After histopathologic analysis, foci of epithelial-myoepithelial carcinoma and poorly differentiated adenocarcinoma (not otherwise specified; NOS) with clear cytoplasm were seen in the parotid gland (Figure 3). Positive surgical margins were seen in the deep lobe of the parotid gland. There was no vascular invasion, perioral invasion, or lymphatic invasion. Immunohistochemical staining showed SMA (+), P63 (+), CK8 (+), and S100 (+) on epithelial cells (Figure 2). Due to the positive surgical margins, the deep lobe of the parotid gland was removed with facial nerve conservation, and functional neck dissection was performed. Postoperative facial nerve functions were normal. Histopathologically, there were no tumor foci in the deep parotid lobe or in the neck-dissection material after the second surgery. The patient was referred to oncology.

Radiotherapy was given to the patient by oncology specialists but chemotherapy was not needed. No recurrence or metastasis has occurred in 3 years of follow-up.

## 3. Discussion

Epithelial-myoepithelial carcinomas are rare low-grade tumors that have biphasic morphology (the integral part consisting of epithelial cells surrounded by myoepithelial cells). They were first described by Roy et al. in 2010 [1]. These tumors are seen in males twice as often as in females, with a peak occurrence in the sixth and seventh decades [2]. In comparison to classic epithelial-myoepithelial carcinoma, dedifferentiated tumors have a worse prognosis and develop more aggressively [2]. The rate of lymph node metastasis is 45% and that of distant metastasis is 36% among dedifferentiated cases [2]. Among epithelial-myoepithelial carcinomas, the rate of lymph node metastasis is 20% and that of distant metastasis is 10% [2]. A review of the literature reveals 22 previously reported cases of dedifferentiated epithelial-myoepithelial carcinoma [4]. In these cases, the malignant tumors dedifferentiated from epithelial-myoepithelial carcinoma were adenoid cystic carcinoma, actinic cell carcinoma, polymorphous low-grade adenocarcinoma, mucoepidermoid carcinoma, and intraductal carcinoma [2, 4]. In our case, the malignant tumor dedifferentiated from an epithelial-myoepithelial carcinoma was a poorly differentiated adenocarcinoma. Metastasis was not seen in the neck-dissection histopathologic material.

Because of limited cases of tumors that are dedifferentiated from epithelial-myoepithelial carcinoma in the literature and lack of proper follow-up, an accurate prognosis is not known. In our patient, there is no local recurrence or metastasis after 3 years of follow-up. We think that this case will make a contribution to the literature in terms of prognosis.

## 4. Conclusion

There is no recurrence or metastasis in our case of dedifferentiated adenocarcinoma from epithelial-myoepithelial carcinoma of the parotid gland after 3 years of follow-up.
